# Salt Sensitive Tet-Off-Like Systems to Knockdown Primordial Germ Cell Genes for Repressible Transgenic Sterilization in Channel Catfish, *Ictalurus punctatus*

**DOI:** 10.3390/md15060155

**Published:** 2017-05-31

**Authors:** Hanbo Li, Baofeng Su, Guyu Qin, Zhi Ye, Ahmed Alsaqufi, Dayan A. Perera, Mei Shang, Ramjie Odin, Khoi Vo, David Drescher, Dalton Robinson, Dan Zhang, Nermeen Abass, Rex A. Dunham

**Affiliations:** 1 School of Fisheries, Aquaculture and Aquatic Sciences, Auburn University, Auburn, AL 36849, USA; hzl0026@tigermail.auburn.edu (H.L.); subaofeng@hrfri.ac.cn (B.S.); gzq0002@tigermail.auburn.edu (G.Q.); zzy0008@tigermail.auburn.edu (Z.Y.); asa0014@tigermail.auburn.edu (A.A.); dperera@wvstateu.edu (D.A.P.); mzs0040@tigermail.auburn.edu (M.S.); ryo0001@tigermail.auburn.edu (R.O.); kmv0005@tigermail.auburn.edu (K.V.); dmd0028@tigermail.auburn.edu (D.D.); dar0037@tigermail.auburn.edu (D.R.); dzz0007@tigermail.auburn.edu (D.Z.); nyy0001@auburn.edu (N.A.); 2 National and Local United Engineering Laboratory for Freshwater Fish Breeding, Heilongjiang River Fisheries Research Institute, Chinese Academy of Fisheries Sciences, Harbin 150070, China; 3 Department of Aquaculture and Animal, King Faisal University, Al Ahsa 31982, Saudi Arabia; 4 Research and Development Corporation, Gus R. Douglass Institute, West Virginia State University, WV 25112, USA; 5 Department of Agricultural Botany, Faculty of Agriculture Saba-Basha, Alexandria University, Alexandria City, P.O. Box 2153, Egypt

**Keywords:** transgenic sterilization, repressible by sodium chloride, PGC migration, RNAi

## Abstract

Repressible knockdown approaches were investigated for transgenic sterilization in channel catfish, *Ictalurus punctatus*. Two primordial germ cell (PGC) marker genes, *nanos* and *dead end*, were targeted for knockdown, and an off-target gene, *vasa*, was monitored. Two potentially salt sensitive repressible promoters, zebrafish *adenylosuccinate synthase 2* (ADSS) and zebrafish *racemase* (Rm), were each coupled with four knockdown strategies: ds-sh RNA targeting the 5′ end (N1) or 3′ end (N2) of channel catfish *nanos*, full-length cDNA sequence of channel catfish *nanos* for overexpression (cDNA) and ds-sh RNA targeting channel catfish *dead end* (DND). Each construct had an untreated group and treated group with sodium chloride as the repressor compound. Spawning rates of full-sibling P_1_ fish exposed or not exposed to the constructs as treated and untreated embryos were 93% and 59%, respectively, indicating potential sterilization of fish and repression of the constructs. Although the mRNA expression data of PGC marker genes were inconsistent in P_1_ fish, most F_1_ individuals were able to downregulate the target genes in untreated groups and repress the knockdown process in treated groups. The results indicate that repressible transgenic sterilization is feasible for reproductive control of fish, but more data from F_2_ or F_3_ are needed for evaluation.

## 1. Introduction

Since the commercialization of AquaBounty Technologies’s AquAdvantage^®^ Salmon [1], the control of escapement of transgenic offspring has received as much attention as the advantages of transgenic fish. A major concern regarding the use of transgenic fish is potential ecological impact from escapees or released individuals on natural populations [2,3]. All transgenic organisms should be adequately confined both physically and genetically to minimize environmental risk.

Physical confinement cannot guarantee that transgenic fish will never establish in the wild since the possibility of theft, human error and catastrophic events could compromise the physical confinement, leading to escape of transgenic animals from aquaculture systems [4]. A genetic confinement system that requires human intervention to allow brood stock production and fertility overcomes these weaknesses. Obviously, the use of both and redundant systems further decreases the risk of establishment of transgenes. If used outside their native geographical range, all species are potentially invasive. Different permutations of transgenic sterilization can potentially be used to prevent spread of invasive species.

The embryonic precursors of the gametes are primordial germ cells (PGCs), their origin is distant from the developmental site of the gonads, and they must migrate to the embryo’s genital ridge [5]. PGCs will eventually die without migration, but the death has no effect on somatic development. As a result, disrupting the migration of PGCs is a potentially viable approach to complete transgenic sterilization. In zebrafish (*Danio rerio*), a number of genetic markers are associated with PGC migration, such as *vasa*, *nanos (nos)*, *askopos*, *dead end* and *dazl*. Knock out of those markers genes can prevent or disrupt the migration of PGCs [6,7,8,9,10]. Prevention of PGC migration invariably produces sterile fish [8,9,10,11,12]. 

*Nanos* is essential for proper migration and survival of PGCs in zebrafish [5,6]. Although the formation of PGCs does not require *nanos* activity, PGCs with *nanos* deficiency demonstrate abnormal conditions such as lack of colonization in the gonad, premature activation of germ cell markers, abnormal morphology and expression of mRNAs that are normally expressed in the soma. *Vasa* is an RNA binding protein with an RNA dependent helicase. Long-term and continual expression in the germ line makes *vasa* a unique PGC marker [5,13,14]. As a PGC marker, the role and functions of *vasa* are not as conserved as other markers as it may have evolved to have different functions in different species, which may explain its longer duration of expression compared to other PGC markers [9,15]. In zebrafish, *vasa* encodes an RNA helicase, and is essential for the assembly of the germplasm and the migration of PGCs [16]. Another function of *vasa* is to overcome the repressive effect of *nanos* translational control element, an evolutionarily conserved dual stem-loop structure in the 3′ UTR, which acts independently of the localization signal to repress translation of *nanos* mRNA [17]. *Dead end* protein is a RNA-binding factor that positively regulates gene expression by prohibiting micro RNA (miRNA)-mediated gene suppression [18]. In zebrafish, miR-430 is a miRNA responsible for the downregulation of several hundred targeted mRNAs in soma cells including *nanos*. By binding to U-rich sequence elements in 3′ UTR of those mRNAs and inhibiting the activity of miR-430, dead end protein protects those mRNAs from degradation in the germ cells, thus, relieving miRNA repression in germline cells by blocking the accessibility of target mRNAs to miRNAs. As the first factor to play a specific role in the initiation of PGC mobility, knockdown of *dead end* terminates the initial migration step of PGCs, dorsal movement within the deep blastoderm [12].

RNAi is frequently used in molecular biotechnology for targeted gene knockdown. Different strategies such as double-stranded RNA (dsRNA), short hairpin RNA (shRNA), miRNA and small interfering RNA (siRNA) can be applied to achieve the goal [19]. When exogenous RNA, which is synthesized with a sequence complementary to a gene of interest, is expressed *in vivo* and the RNAi pathway activated, the target gene is silenced [20]. Unlike the use of dsRNA and shRNA for RNAi, overexpression of a target gene via full-length cDNA is another option to inhibit target genes. This process should trigger a negative feedback loop or have undesired malicious effects, leading to disruption of the stoichiometry of the protein complex and loss of function. In the case of the negative feedback loop, the excess *nanos* cDNA would be detected and the response would be the interruption of *nanos* mRNA transcription or protein translation. The effect of the response is the downregulation of *nanos* expression or degradation of *nanos* protein, resulting in *nanos* knockdown.

Function of the *bone morphogenetic protein 2* (BMP-2) gene in zebrafish, common carp and channel catfish has been successfully disrupted using the cDNA overexpression approach [21]. Bernick et al. [22] overexpressed Unc-45b in zebrafish embryos, which resulted in defective thick filament organization. Overexpression could complement loss-of-function screens, and has dominant effects [23].

Modified Tet-off system has been applied to produce repressible transgenic sterile fish and oysters [21], although these systems have yet to be shown 100% effective or commercially feasible. Theoretically, the Tet-off system should be an effective repressible sterility system for preventing the establishment of feral populations of transgenic animals or exotic species [24]; however, the antibiotic repressors used in the system, tetracycline and doxycycline, are expensive when used in large-scale applications. In addition, accumulation or release of large amounts of antibiotics may result in antibiotic resistance of pathogens in the aquaculture environment, as well as the presence of antibiotics in drinking water. Additionally, the Tet-off transgenes contain small viral sequences that might be negatively perceived by consumers. Environmentally friendly and efficient Tet-off-like systems are needed, and promoters that can be repressed by non-antibiotic chemicals are preferred in the new systems.

Sodium chloride based Tet-off like systems could be such a system. Channel catfish embryos can tolerate 6 ppt of salinity in hatchery solutions [25], sodium chloride is commonly used in catfish culture to control disease and address water quality problems such as high nitrite [26]. It is inexpensive, easy to apply and when used in small quantities would cause no environmental damage. Thus, salt sensitive promoters could be viable replacements in Tet-off-like system, such as zebrafish *adenylosuccinate synthase 2* (*adss2*) and *racemace.* These promoters were salt sensitive and can be repressed by 4 ppt NaCl in the laboratory [27], and thus, may be very appropriate for repressible sterilization in freshwater fish.

The knockdown constructs driven by a salt-sensitive promoter could be introduced into the fish. The PGC knockdown construct expresses, prevents PGC migration, and thus, sterilizes embryos not exposed to salt. These would be the production fish that are sterile and cannot reproduce if they were to escape into the natural environment. A portion of the embryos would be treated with salt, which should repress the knockdown construct and allow normal sexual development for the development of brood stock. If these fish escape, they can only produce sterile offspring, thus the transgene cannot become established in the receiving population and is present for one generation or less.

The objectives of the this study were to use transgenic technology to prevent PGC migration and gamete formation, leading to sterility in channel catfish, and then repress this process to produce fertile brood stock that generate sterile offspring. Specifically, zebrafish *adss2* and *racemase* promoters were evaluated to control the expression of ds-shRNA or cDNA constructs to knockdown targeted PGC migration related genes, *nanos* and *dead end*, and their repression evaluated by dosing with sodium chloride, towards the ultimate objective of successful repressible transgenic sterilization. Spawning success was measured in treated and untreated P_1_ brood stock, and PGC marker gene expression was determined in salt treated and untreated P_1_ and F_1_ generations.

## 2. Results

### 2.1. Expression of Nanos, Vasa and Dead End in Channel Catfish Embryos

**Figure 1:**
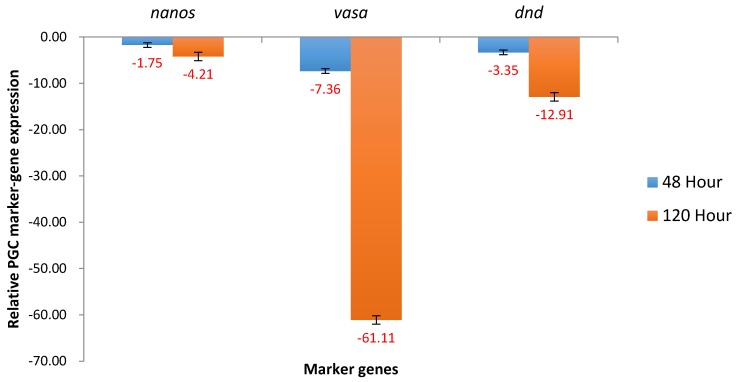
Relative expression of primordial germ cell (PGC) marker genes, *nanos*, *vasa*, and *dead end* (*dnd*) in non-transgenic channel catfish (*Ictalurus punctatus*) embryos. The samples were analyzed at 24, 48 and120 h post fertilization (hpf). Relative *nanos*, *vasa* and *dnd* genes expression at 48 hpf and 120 hpf were expressed as fold change over 24 hpf sample as normalized to change in the expression of 18 s control. All the red fold values numbers shown were significant at the level of *p* < 0.05 using Pairwise Fixed Reallocation Randomization Test (PFRR) from REST (Relative Expression Software Tool).

At 48 and 120 hours post fertilization (hpf), *nanos* expression was declining relatively slowly, −1.75X and −4.21X, respectively, relative to expression levels present at 24 hpf. The level of *vasa* was 7.36X lower at 48 hpf than at 24 hpf, and was dramatically lower, −61.11X at 120 hpf compared to 24 hpf. *Dead end* was downregulated 3.35X at 48 hpf and 12.91X at 120 hpf compared to 24 hpf (Figure 1).

### 2.2. P_1_ Spawning

The overall spawning rates were significantly different (*p* < 0.05) for pooled untreated and treated groups, 59% and 93% (Table 1), respectively. P_1_ fish are always mosaic by tissue and by cells within tissues. When exogenous DNA is introduced to fish embryos, it replicates and is present in the cytoplasm for a long period during development before it is degraded. Therefore, transgenes that influence embryonic traits can have a phenotypic effect on the F_1_ through exposure alone and do not necessarily need to be integrated for proof of principle. We have unpublished data, as well as published data [21], indicating phenotypic change in embryonic traits by exposure (electroporating) of the embryos to transgenes. Transfection rates are typically 20–50% [28,29] in P_1_.

Fish from three, ADSSN1, ADSSN2 and RMDND, of the eight constructs in the untreated group had spawning rates lower than 50%, while females from five of the eight constructs in the treated group had 100% spawning rates. There were significant differences (*p* < 0.05) between treated and untreated groups, for ADSSN2 construct as well as in pooled groups of all constructs.

When pooled by promoters, spawning rates in untreated groups and treated groups from the ADSS system had significant differences (*p* < 0.05) between transgenic treated and untreated groups. The ratio of treated spawning rate to untreated spawning rate was 1.94 (Table 2).

When pooling by knockdown strategy, spawning rates for untreated and treated groups of N2 had the best repression/knockdown efficiency compared to other strategies, with the efficiency ratio of 1.72 (Table 2).

### 2.3. Real-Time Quantitative PCR Results for the P_1_ and F_1_ Generation Transgenic Channel Catfish

#### 2.3.1. ADSS System

**Figure 2:**
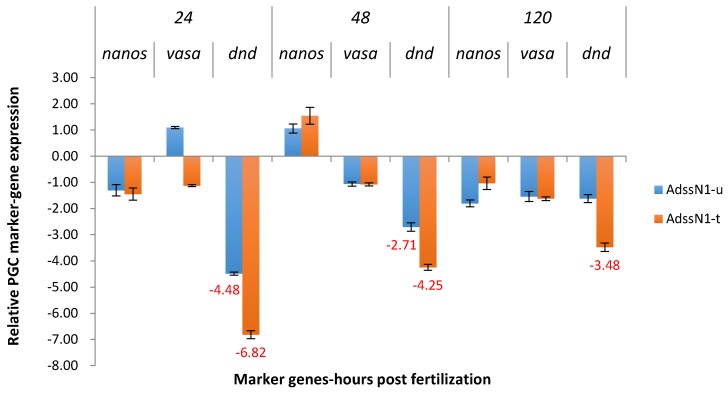
Relative expression of primordial germ cell (PGC) marker genes, *nanos, vasa,* and *dead end* (*dnd*) in the treated and untreated groups exposed to zebrafish *adenylosuccinate synthase 2* (*adss2*) gene (ADSS) promoter fused with 5’ end shRNAi targeting channel catfish *nanos* gene (N1) as P_1_ ADSSN1 transgenic channel catfish (*Ictalurus punctatus*) embryos. *u* represents untreated group, *t* represents treated group. The treated group was incubated in 4 ppt sodium chloride. The samples were analyzed at 24, 48 and 120 h post fertilization (hpf), respectively. Relative *nanos, vasa,* and *dead end (dnd)* gene expression was expressed as fold change over non-transgenic control samples at the same time point. All the red fold values numbers shown were significantly different compared to non-transgenic control at the level of *p* < 0.05 using Pairwise Fixed Reallocation Randomization Test (PFRR) from REST (Relative Expression Software Tool). Red X on the SE bar indicated the relative expression between the treated and untreated groups was significantly different from each other using PFRR.

*Nanos* of ADSSN1 was not significantly downregulated in P_1_ (Figure 2), for both treated and untreated groups. Treated and untreated groups showed similar off-target patterns of decreasing expression of *dead end,* from 6.82X to 3.48X for salt treated and 4.48X to 1.62X for the untreated. Differences between the salt treated and untreated group were minimal and inconsistent. There were no data generated for ADSSN1 in the F_1_ generation as no females were available to spawn in the treated group and the embryos from the untreated group were not transgenic (Table 2).

**Figure 3:**
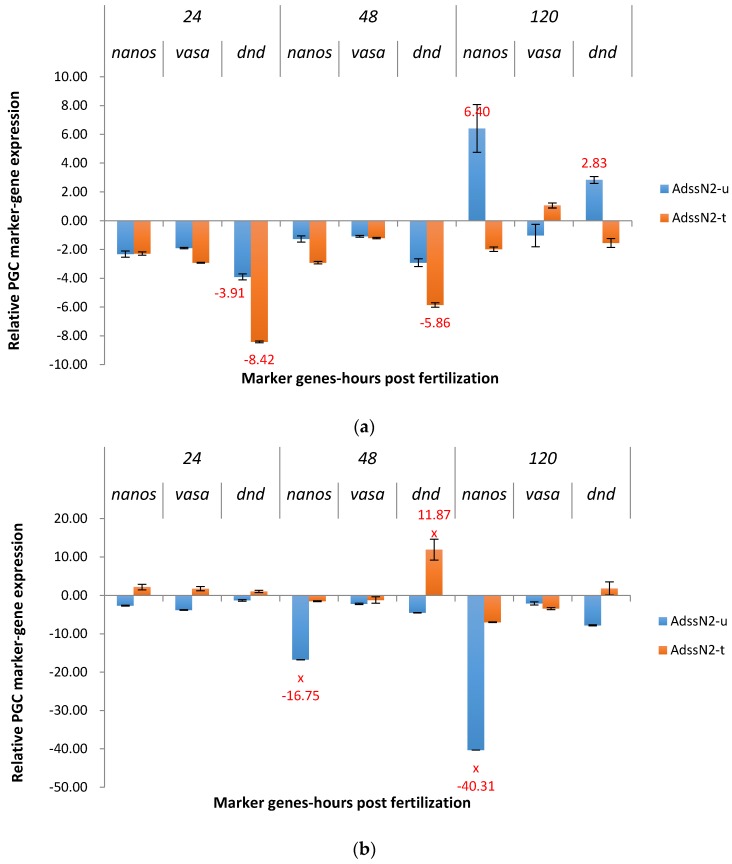
Relative expression of primordial germ cell (PGC) marker genes, *nanos*, *vasa*, and *dead end* (*dnd*) in the treated and untreated groups exposed to zebrafish *adenylosuccinate synthase 2* (*adss2*) gene (ADSS) promoter fused with 3′ end shRNAi targeting channel catfish *nanos* gene (N2) as P_1_ (**a**) and F_1_ (**b**) ADSSN2 transgenic channel catfish (*Ictalurus punctatus*) embryos. *u* represents untreated group, *t* represents treated group. The treated group was incubated in 4 ppt sodium chloride. The samples were analyzed at 24, 48 and 120 h post fertilization (hpf), respectively. Relative *nanos*, *vasa*, and *dead end* (*dnd*) gene expression was expressed as fold change over non-transgenic control samples at the same time point. All the red fold values numbers shown were significantly different compared to non-transgenic control at the level of *p* < 0.05 using Pairwise Fixed Reallocation Randomization Test (PFRR) from REST (Relative Expression Software Tool). Red X on the SE bar indicated the relative expression between the treated and untreated groups was significantly different from each other using PFRR.

ADSSN2 knocked down *nanos* in the P_1_ treated group from 1.98X to 2.91X at three time points (Figure 3a). Untreated ADSSN2 had variable effects on *nanos* at different time points as there was downregulation for *nanos* (2.32X) at 24 hpf, and upregulation for *nanos* (6.40X) at 120 hpf. Similar to ADSSN1, a strong off-target effect was observed in the treated group for knockdown *dead end*.

F_1_ ADSSN2 treatments yielded results as hypothesized. *Nanos* was significantly downregulated for 16.75X at 48 hpf and 40.31X at 120 hpf (Figure 3b), in untreated group. Most time points from the treated group had similar expression patterns as the non-transgenic control, except the off-target *dead end* at 48 hpf, which was significantly upregulated for 11.87X.

**Figure 4:**
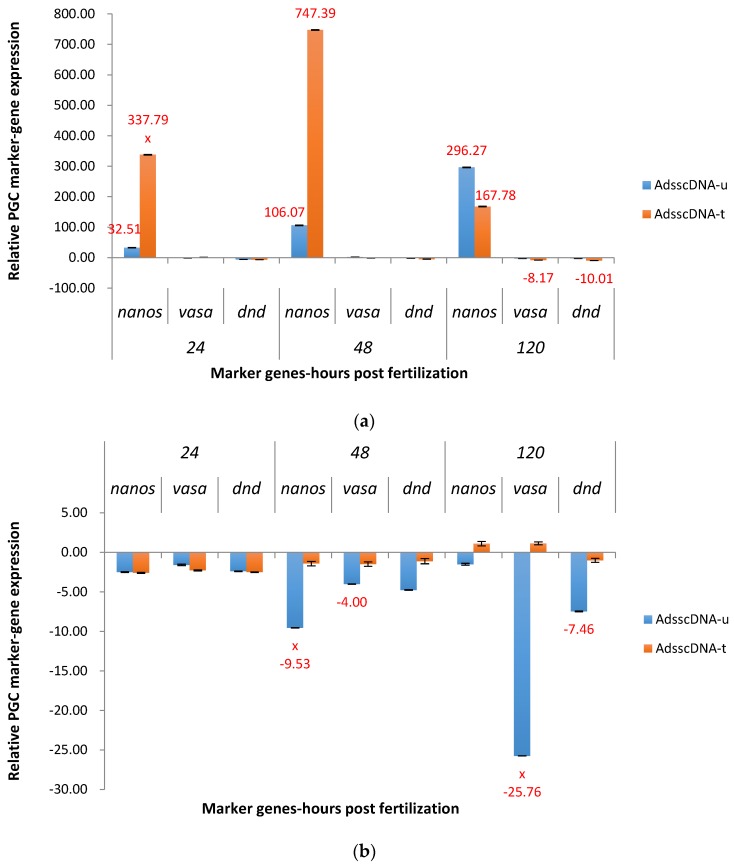
Relative expression of primordial germ cell (PGC) marker genes, *nanos*, *vasa*, and *dead end* (*dnd*) in the treated and untreated groups exposed to zebrafish *adenylosuccinate synthase 2* (*adss2*) gene (ADSS) promoter fused with full-length cDNA of channel catfish *nanos* gene (cDNA) as P_1_ (**a**) and F_1_ (**b**) ADSScDNA transgenic channel catfish (*Ictalurus punctatus*) embryos. *u* represents untreated group, *t* represents treated group. The treated group was incubated in 4 ppt sodium chloride. The samples were analyzed at 24, 48 and 120 h post fertilization (hpf), respectively. Relative *nanos, vasa,* and *dead end (dnd)* gene expression was expressed as fold change over non-transgenic control samples at the same time point. All the red fold values numbers shown were significantly different compared to the non-transgenic control at the level of *p* < 0.05 using Pairwise Fixed Reallocation Randomization Test (PFRR) from REST (Relative Expression Software Tool). Red X on the SE bar indicated the relative expression between the treated and untreated groups was significantly different from each other using PFRR.

Both ADSScDNA treated and untreated P_1_ strongly upregulated the expression of *nanos* gene at three time points (Figure 4a). In the treated group, upregulation of *nanos* varied from 167.78X to 747.39X at the three time points, and the peak value was at 48 hpf. In the untreated group, the upregulation of *nanos* was from 32.51X (24 hpf) to 296.27X (120 hpf). *Dead end* was down regulated in both treated and untreated groups from 2X to 10X at three time points. *Vasa* and *dead end* was downregulated 8.17X and 10.01X at 120 hpf in the treated group.

For the F_1_ generation, ADSScDNA downregulated *nanos* at three time points in the untreated groups (Figure 4b), especially at 48 hpf (9.53X). Fish with this construct were consistently downregulated for *vasa*. At 24 hpf, *nanos*, *vasa* and *dead end* were downregulated in a similar fashion in both treated and untreated groups.

**Figure 5:**
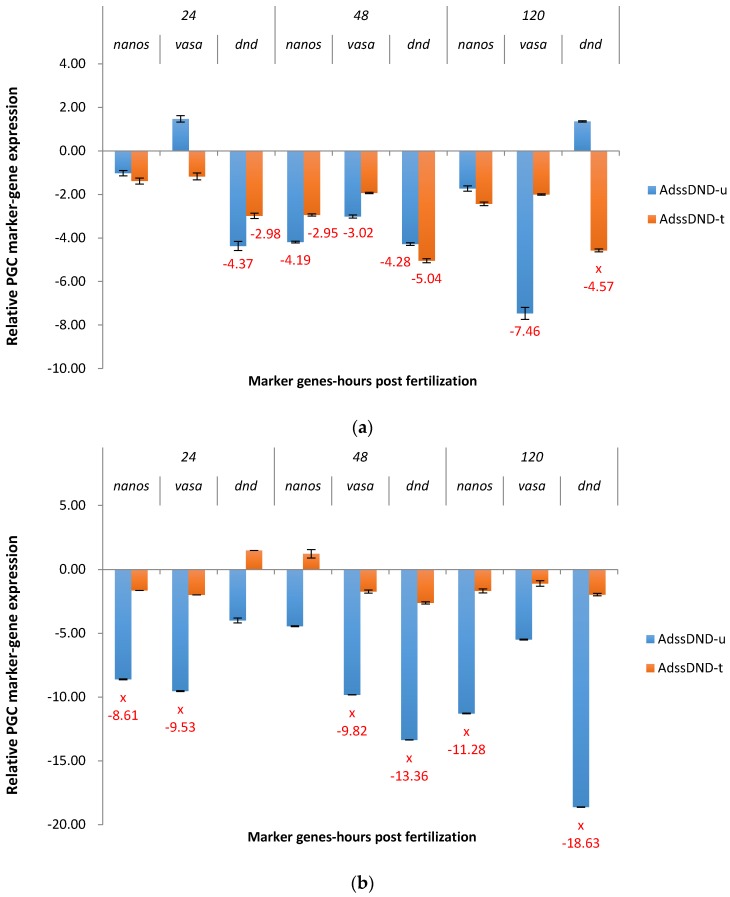
Relative expression of primordial germ cell (PGC) marker genes, *nanos*, *vasa*, and *dead end* (*dnd*) in the treated and untreated groups exposed to zebrafish *adenylosuccinate synthase 2* (*adss2*) gene (ADSS) promoter fused with shRNAi targeting channel catfish *dead end* gene (DND) as P_1_ (**a**) and F_1_ (**b**) ADSSDND transgenic channel catfish (*Ictalurus punctatus*) embryos. *u* represents the untreated group, *t* represents treated group. The treated group was incubated in 4 ppt sodium chloride. The samples were analyzed at 24, 48 and 120 h post fertilization (hpf), respectively. Relative *nanos*, *vasa*, and *dead end* (*dnd*) gene expression was expressed as fold change over non-transgenic control samples at the same time point. All the red fold values numbers shown were significantly different compared to the non-transgenic control at the level of *p* < 0.05 using Pairwise Fixed Reallocation Randomization Test (PFRR) from REST (Relative Expression Software Tool). Red X on the SE bar indicated the relative expression between the treated and untreated groups was significantly different from each other using PFRR.

For P_1_ ADSSDND exposed channel catfish, *dead end* was downregulated from 2.98X to 5.04X, at three time points in treated groups, and at 24 hpf and 48 hpf in untreated group (Figure 5a). At 120 hpf, *dead end* was significantly up regulated in the untreated group. Strong off-target effects were observed: *vasa* expression was knocked down at 48 and 120 hpf in the untreated group, from 3.02X to 7.46X.

The untreated F_1_ ADSSDND group was significantly downregulated for *dead end* at 48 hpf (13.36X) and 120 hpf (18.63X) (Figure 5b). Significant off-target downregulation was observed for *nanos and vasa* in the untreated group at all three time points, especially at 24 hpf (8.61X for *nanos* and 9.53X for *vasa*), 48 hpf (9.82X for *vasa*), and 120 hpf (11.28X for *nanos*).

#### 2.3.2. RM System

**Figure 6:**
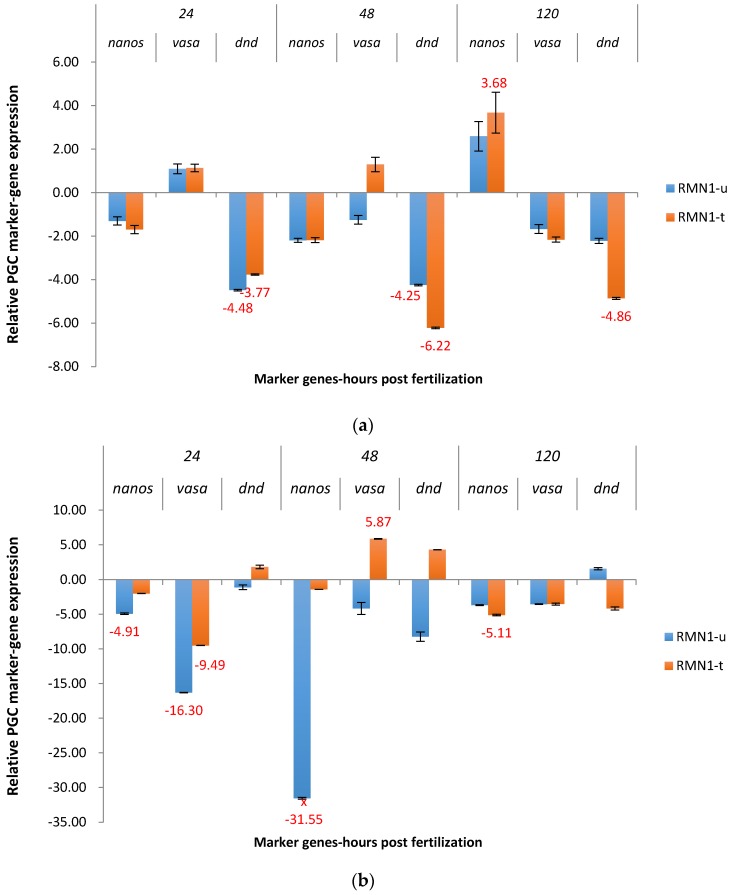
Relative expression of primordial germ cell (PGC) marker genes, *nanos, vasa,* and *dead end (dnd)* in the treated and untreated groups exposed to zebrafish *racemase* gene (RM) promoter fused with 5′ end shRNAi targeting channel catfish *nanos* gene (N1) as P_1_ (**a**) and F_1_ (**b**) RMN1 transgenic channel catfish (*Ictalurus punctatus*) embryos. *u* represents untreated group, *t* represents treated group. The treated group was incubated in 4 ppt sodium chloride. The samples were analyzed at 24, 48 and 120 h post fertilization (hpf), respectively. Relative *nanos*, *vasa*, and *dead end* (*dnd*) gene expression was expressed as fold change over non-transgenic control samples at the same time point. All the red fold values numbers shown were significantly different compared to the non-transgenic control at the level of *p* < 0.05 using Pairwise Fixed Reallocation Randomization Test (PFRR) from REST (Relative Expression Software Tool). Red X on the SE bar indicated the relative expression between the treated and untreated groups was significantly different from each other using PFRR.

*Nanos* was slightly downregulated in P_1_ embryos electroporated with RMN1 construct at 24 hpf and 48 hpf in both treated and untreated groups (Figure 6a). At 120 hpf, *nanos* was upregulated in treated and untreated groups, 3.68X and 2.58X, respectively. The off-target *dead end* was downregulated at three time points in both groups, from 2.11X to 6.22X.

In untreated F_1_ RMN1 channel catfish embryos, *nanos* was downregulated from 3.54X to 31.55X at three points, while in the treated group, the expression of *nanos* was similar to the non-transgenic control, except at 120 hpf (Figure 6b). In addition, off-target downregulation occurred for *vasa* at 24 hpf (16.30X) in the untreated group and 9.49X in the treated group.

**Figure 7:**
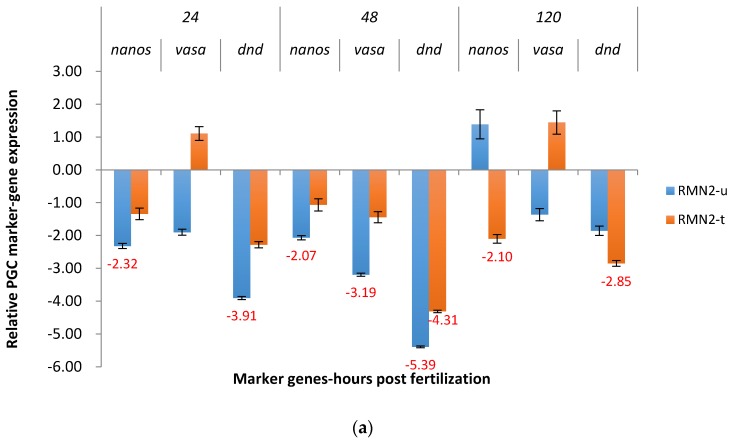

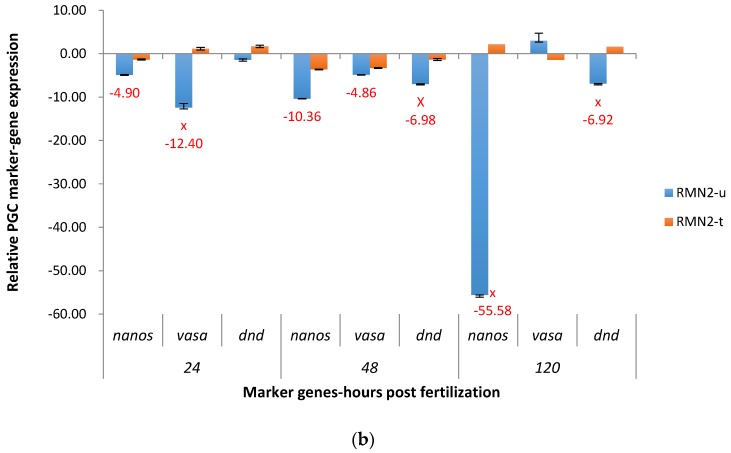
Relative expression of primordial germ cell (PGC) marker genes, *nanos*, *vasa*, and *dead end* (*dnd*) in the treated and untreated groups exposed to zebrafish *racemase* gene (RM) promoter fused with 3′ end shRNAi targeting channel catfish *nanos* gene (N2) as P_1_ (**a**) and F_1_ (**b**) RMN2 transgenic channel catfish (*Ictalurus punctatus*) embryos. *u* represents untreated group, *t* represents treated group. The treated group was incubated in 4 ppt sodium chloride. The samples were analyzed at 24, 48 and 120 h post fertilization (hpf), respectively. Relative *nanos*, *vasa*, and *dead end* (*dnd*) gene expression was expressed as fold change over non-transgenic control samples at the same time point. All the red fold values numbers shown were significantly different compared to the non-transgenic control at the level of *p* < 0.05 using Pairwise Fixed Reallocation Randomization Test (PFRR) from REST (Relative Expression Software Tool). Red X on the SE bar indicated the relative expression between the treated and untreated groups was significantly different from each other using PFRR.

At 24 hpf and 48 hpf, untreated P_1_ embryos exposed to RMN2 were downregulated for *nanos*, 2.32X and 2.07X (Figure 7a). The salt treatment repressed downregulation of *nanos* at 24 and 48 hpf, but not at 120 hpf (2.10X). Off-target downregulation, 1.85X to 5.39X, was observed for *dead end* in both groups at three time points. The differences in expression between salt treated and untreated embryos at the same time point were minimal. At 48 hpf, *vasa* was also significantly downregulated, 3.19X, in the untreated group.

In the case of F_1_ RMN2 channel catfish embryos, *nanos* was significantly downregulated at three time points in the untreated group (4.90X to 55.58X) (Figure 7b). For salt treated groups, downregulation of *nanos* was successfully repressed. Off-target effects occurred as *vasa* and *dead end* also had decreased expression, at 24 hpf and 48 hpf for *vasa*, 48 hpf and 120 hpf for *dead end* in the untreated group.

**Figure 8:**
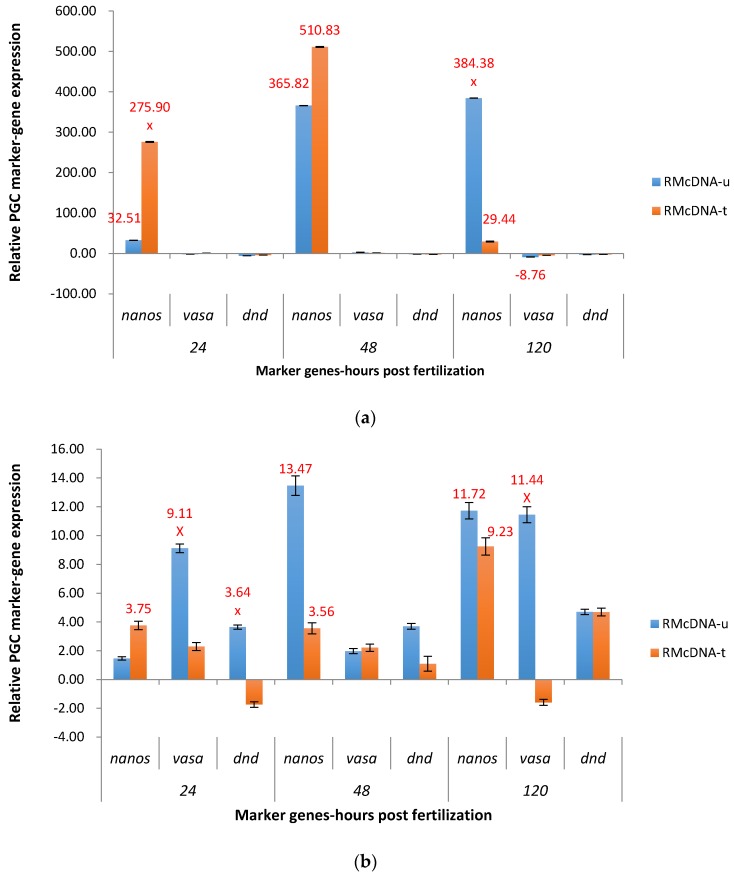
Relative expression of primordial germ cell (PGC) marker genes, *nanos*, *vasa*, and *dead end* (*dnd*) in the treated and untreated groups exposed to zebrafish *racemase* gene (ADSS) promoter fused with full-length cDNA of channel catfish *nanos* gene (cDNA) as P_1_ (**a**) and F_1_ (**b**) RMcDNA transgenic channel catfish (*Ictalurus punctatus*) embryos. *u* represents untreated group, *t* represents treated group. The treated group was incubated in 4 ppt sodium chloride. The samples were analyzed at 24, 48 and 120 h post fertilization (hpf), respectively. Relative *nanos*, *vasa*, and *dead end* (*dnd*) gene expression was expressed as fold change over non-transgenic control samples at the same time point. All the red fold values numbers shown were significantly different compared to the non-transgenic control at the level of *p* < 0.05 using Pairwise Fixed Reallocation Randomization Test (PFRR) from REST (Relative Expression Software Tool). Red X on the SE bar indicated the relative expression between the treated and untreated groups was significantly different from each other using PFRR.

Upregulation of *nanos* in P_1_ RMcDNA electroporated embryos was significant, 29.44X (120 hpf) to 510.83X (48 hpf) in both groups (Figure 8a). In the untreated group, the upregulation of *nanos* was continuously increasing from 32.51X (24 hpf) to 384.38X (120 hpf), and in the treated group, the upregulation started at 275.90X at 24 hpf, reached its peak of 510.83X at 48 hpf, and then decreased to 29.44X at 120 hpf.

For F_1_ embryos in the untreated RMcDNA group, *nanos* was significantly upregulated at 48 hpf and 120 hpf, 13.47X and 11.72X, respectively (Figure 8b). In the salt treated group, the upregulation of *nanos* was largely repressed compared to untreated at 48 hpf (downregulation of 3.56X), but not at 120 hpf (9.23X). Strong off-target effects for *vasa* were apparent at 24 hpf (upregulation of 9.11X) and 120 hpf (upregulation of 11.44X) for the untreated group.

**Figure 9:**
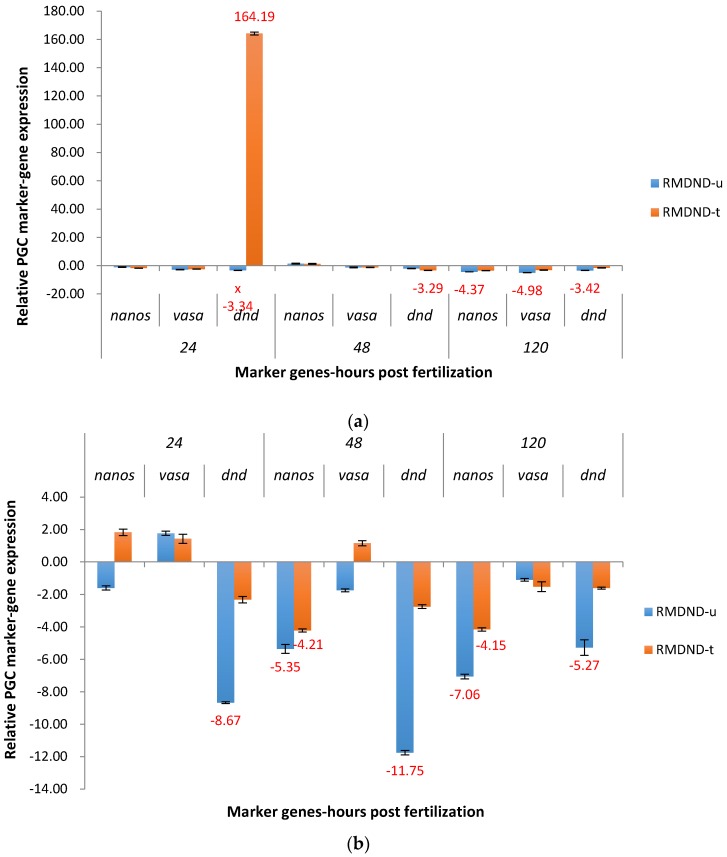
Relative expression of primordial germ cell (PGC) marker genes, *nanos*, *vasa*, and *dead end* (*dnd*) in the treated and untreated groups exposed to zebrafish *racemase* gene (RM) promoter fused with shRNAi targeting channel catfish *dead end* gene (DND) as P_1_ (**a**) and F_1_ (**b**) RMDND transgenic channel catfish (*Ictalurus punctatus*) embryos. *u* represents untreated group, *t* represents treated group. The treated group was incubated in 4 ppt sodium chloride. The samples were analyzed at 24, 48 and 120 h post fertilization (hpf), respectively. Relative *nanos*, *vasa*, and *dead end* (*dnd*) gene expression was expressed as fold change over non-transgenic control samples at the same time point. All the red fold values numbers shown were significantly different compared to the non-transgenic control at the level of *p* < 0.05 using Pairwise Fixed Reallocation Randomization Test (PFRR) from REST (Relative Expression Software Tool). Red X on the SE bar indicated the relative expression between the treated and untreated groups was significantly different from each other using PFRR.

*Dead end* was downregulated, 1.71X to 3.42X, for RMDND in both P_1_ groups at three time points except in treated group at 24 hpf (upregulated 164.19X) (Figure 9a). Additionally, *vasa* and *nanos* were significantly knocked down at 120 hpf in the untreated group (4.98X and 4.37X).

The target gene *dead end* was significantly downregulated, 5.27X to 11.75X, in the F_1_ untreated RMDND group at three time points (Figure 9b). In the treated group, downregulation of *dead end* was rapidly reduced at each time point compared to the untreated group to a level of −1.60X to −2.74X. *Nanos* was significantly downregulated in both groups at 48 hpf and 120 hpf, indicative of a strong off-target effect or gene interaction.

## 3. Discussion

Knockdown constructs using shRNAi and overexpression of cDNA that targeted the PGC genes, *nanos* and *dead end*, which play important roles in PGC migration, were electroporated into channel catfish to achieve transgenic sterilization. Subsequently, F_1_ individuals were produced containing the knockdown constructs. These constructs were designed to be repressed by application of salt to accomplish repressible transgenic sterilization. Gene expression of PGC marker genes during the early development of P_1_ electroporated individuals, thus exposed to the constructs during putative PGC migration, that were either treated with 4 ppt sodium chloride or untreated indicated that, in some cases, the constructs were able to downregulate the targets or overexpress the target genes, but the repression was not very effective or in some cases, actually enhanced down or upregulation. The results were different for F_1_ transgenic individuals, as sterilization constructs were downregulating the key PGC genes, and that the repression was generally successful. However, the cDNA overexpression constructs did not work as expected with the ADSS driven construct reducing mRNA levels, but the RM driven construct dramatically increasing the mRNA levels. Many complementary off-target effects on the related PGC genes were observed. Spawning data on P_1_ treated and untreated brood stock exposed to the transgenes as embryos supported the hypothesis that the repressible transgenic system based on sodium chloride sensitive promoters is workable as spawning rates of the sodium chloride treated fish was higher than the untreated. 

In the case of control channel catfish embryos that had not been exposed to the transgenes, levels of *nanos* mRNA dropped naturally from 24 to 120 hpf, *dead end* dropped slightly faster than *nanos*, and *vasa* was dramatically lower, at 120 hpf compared to 24 hpf. Either the expression of mRNA of those three PGC migration marker genes was downregulated naturally or there was maternally derived mRNA of those marker genes in the early phase of embryonic development, which was being degraded later in embryogenesis. However, maternally derived *nanos* mRNA in zebrafish degrades rapidly before gastrulation [6], and Xu et al. [31] have shown that during embryogenesis in Asian seabass (*Lates calcarifer*), the *vasa* transcript is abundant in early stages, and persists at a reduced and detectable level in late stages. This supports the premise that the decreases in the *nanos, vasa*, and *dead end* mRNA levels in channel catfish were due to natural and transgenic downregulation, and not just degradation.

These observations on embryonic expression levels of *nanos, vasa*, and *dead end* mRNA might have implications for different knockdown strategies to prevent migration of PGCs and their potential success in channel catfish and other fish species [32]. Understanding the mRNA regulation pattern of the target genes would help us better explain the knockdown results at different time points. A more detailed study of the expression level of these three mRNAs in embryonic development of channel catfish might be beneficial for identifying the transgenic sterilization constructs with the best probability of success and further refinement of the repressible transgenic sterilization systems.

Previous data using copper sensitive promoters in zebrafish and channel catfish for repressible sterilization on P_1_ embryos have shown that they were able to drive transcription in channel catfish and common carp embryos that had been electroporated with the knockdown constructs and likely had large copy numbers in their cytoplasm [32,33]. The expression data of copper systems on the PGC targets of the F_1_ transgenic embryos that had integrated copies of the repressible transgenic sterilization constructs was in general agreement with the results from the P_1_ (unpublished data). The salt and copper systems both show good potential for repressible transgenic sterilization, and both present viable options for further development of this concept.

The knockdown/overexpression and repression efficiency were quite different between P_1_ and F_1_ samples. Based on previous unpublished research from our laboratory, we predicted that the embryonic exposure to the constructs in the P_1_ generation would be indicative of results in the F_1_, but they did not correlate well. The P_1_ expression data did show that the constructs were capable of downregulation, but the repression appeared problematic. The downregulation in the F_1_ transgenic embryos was often greater than their parental embryos that were exposed to the foreign constructs via electroporation, and the repression often more efficient in F_1_ embryos. Another difference between P_1_ and F_1_ generations was that in ADSScDNA and RMcDNA, *nanos* was dramatically upregulated in the parental generation, but the upregulation was repressed or reversed in F_1_. 

There are several potential explanations for the differing results observed between the P_1_ and F_1_. P_1_ were likely a mixture of both transgenic and non-transgenic embryos as integration rates would likely between 30% and 70% [28]. We expect that exposure to the constructs should cause sterilization if they are expressing, however, the percentage of embryos for which the electroporation was successful and how many molecules penetrated the developing embryos is unknown. Expression of positive or exposed embryos could be partially masked by those that are negative or for those that the electroporation was not successful. These results also show that although data on P_1_ for embryonic traits generated from exposure to transgenes has some value and relationship to what might be obtained in integrated F_1_ individuals, the relationship may not be perfectly predictive. Results for P_1_ and F_1_ common carp exposed or containing a shRNAi targeting aromatase were contradictory [34]. However, in this case, usefulness of the P_1_ data may have been more compromised, as the trait in question, gender, is determined several days after hatching.

Strong off-target effects for related PGC genes were common in both P_1_ and F_1_ generations. The off-target effects might be caused by mismatch of siRNA [35,36]. In addition, there might be strong genetic interaction effects among *nanos*, *vasa* and *dead end* (Table 3). *Dead end* protein acting to counteract the inhibitory function of several miRNAs, thereby allowing the expression of PGC specific proteins such as *nanos* and *tdrd7* [18,37]. In *Drosophila*, the *vasa* gene encodes an ATP-dependent RNA helicase of the DEAD-box family and was required for promoting translation of at least two known mRNAs, *nanos* and *gurken* [38]. *Dazl* stimulates *vasa* translation by binding to the 3′ UTR of *vasa* mRNA in vivo, *Dazl* knockout mice have reduced levels of *vasa* protein [39]. In mouse, *nanos2* post-transcriptionally represses *Dazl* mRNA expression in germ cells [40], therefore indirectly regulate the expression of *vasa*. In human, *dnd1* have strong interaction score with *dazl* by prediction [41]. Consistent with this observation, ADSSDND targeted *dead end,* but also downregulated *nanos* and *vasa*, at all three time points; knockdown of *nanos* in RMN2 also downregulate *vasa* and *dead end* at different time points. Although there was no evidence of *nanos* affecting the expression of *dead end*, it is possible there are undiscovered direct or indirect interactions between them. If the off-target effects were restricted to PGC migration loci, they may contribute to sterility. 

In F_1_ generation, knockdown and repression in most constructs were much closer to what was expected compared to the results from their parents. In untreated groups, the knockdown constructs were able to significantly downregulate the mRNA expression of the corresponding target gene. In treated groups, sodium chloride treatment repressed expression of knockdown constructs and the mRNA levels of the target genes were near normal. The following constructs, ADSSN2, ADSSDND, RMN2, RMDND, had the best patterns of downregulation with effective repression, followed by RMN1 (not at all timepoints) and ADSScDNA (strong off-target effects with repression). These results are even more encouraging, considering we usually obtain an inheritance rate of 20–50% for the F1 generation. Thus, one-half or less of the embryos in the pooled positive samples are likely transgenic. Significantly different expression levels, downregulation, were obtained even though there were non-transgenic individuals mixed in with the transgenic embryos.

In some cases, knockdown for the target gene was inconsistent at different embryonic stages evaluated. It is not known exactly when the critical period of PGC marker gene knockdown is that ensures the PGC does not reach the genital ridge or dies. Based on our results, it appears it is not necessary to knockdown the PGC gene expression during all of embryogenesis to achieve sterilization since in some cases knockdown was only achieved during one time period, yet P_1_ still had a reduced rate of reproduction. However, further research is necessary to confirm these apparent results, and to further refine the repressible sterilization system. 

With respect to spawning, ADSSN2 was the best system (treated P_1_ with higher spawning rate than untreated). In general, the repression was quite effective, as the treated brood stock had 93% spawning rate for all treatments pooled compared to 59% spawning rate for the untreated brood stock. To meet the goal of eliminating environmental risk and preventing introgression of the transgene into wild populations, the sterilization likely need to be 100% effective, and would likely be a requirement of regulatory agencies. Biologically, an effectiveness of less than 100% would likely still achieve the goal of the transgenes not establishing unless they had a selective advantage. This rate of sterilization in the P_1_ was not achieved, which was not surprising because of the expected mosaicism in P_1_ transgenic fish and the fact that electroporation may not have been successful for each embryo. The observed spawning differences between the treated and untreated P_1_ brood stock indicate that this salt-based sterilization and repression system has good potential. F_1_ spawning data will be needed to determine if this system can be 100% effective. Based upon preliminary, unpublished results with related constructs, we predict 100% sterility of the F_1_ with the salt-sensitive constructs.

Four ppt sodium chloride was used as the repressor. Future research is needed regarding the minimum doses needed to repress the knockdown constructs. This is especially important as if the promoters are too sensitive and can be repressed at low, but naturally found levels of sodium chloride, escaped transgenic channel catfish could establish in the wild, negating the usefulness of the sterilization constructs evaluated in the current study. Channel catfish and blue catfish, *I. furcatus*, have spawned on rare occasion at salinity levels as high as 2 ppt [42]. For the vast majority of the geographic range of channel catfish, the salinity is less than 100 ppm. Likely, ADSS and RM promoters need to not be sensitive to 2.5 ppt sodium chloride and lower for this transgenic sterilization system to be commercially applicable. Otherwise, escaped catfish transgenic for performance traits stacked with the salt sterilization constructs could spawn if they encountered partially saline water in estuarine environments that repressed the construct. If these constructs were selectively advantageous, the transgenes could become established in the natural environment with possible positive, negative or neutral impacts on the ecosystem. If these promoters are repressed at ≤2.5 ppt, it might be possible to alter them to be less sensitive to salt to make them usable in the entire geographic range of channel catfish to prevent establishment of transgenic channel catfish.

## 4. Materials and Methods

All investigations and experimental studies on animals were conducted according to Institutional Animal Care and Use Committees (IACUC) and Association for Assessment and Accreditation of Laboratory Animal Care (AAALAC) protocols and guidelines.

### 4.1. Construction of Plasmids

FRMwg plasmid [30] was used as the vector for all the transgene constructs in this experiment, and it had three functional components: insulator, ocean pout (*Zoarces americanus*) terminator and boundary element. Between these components, there were two salt sensitized promoters that activate the expression of knockdown constructs: a 577 bp zebrafish *adss2* (ADSS) promoter and a 565 bp zebrafish *racemase* (RM) promoter. 

**Figure 10:**
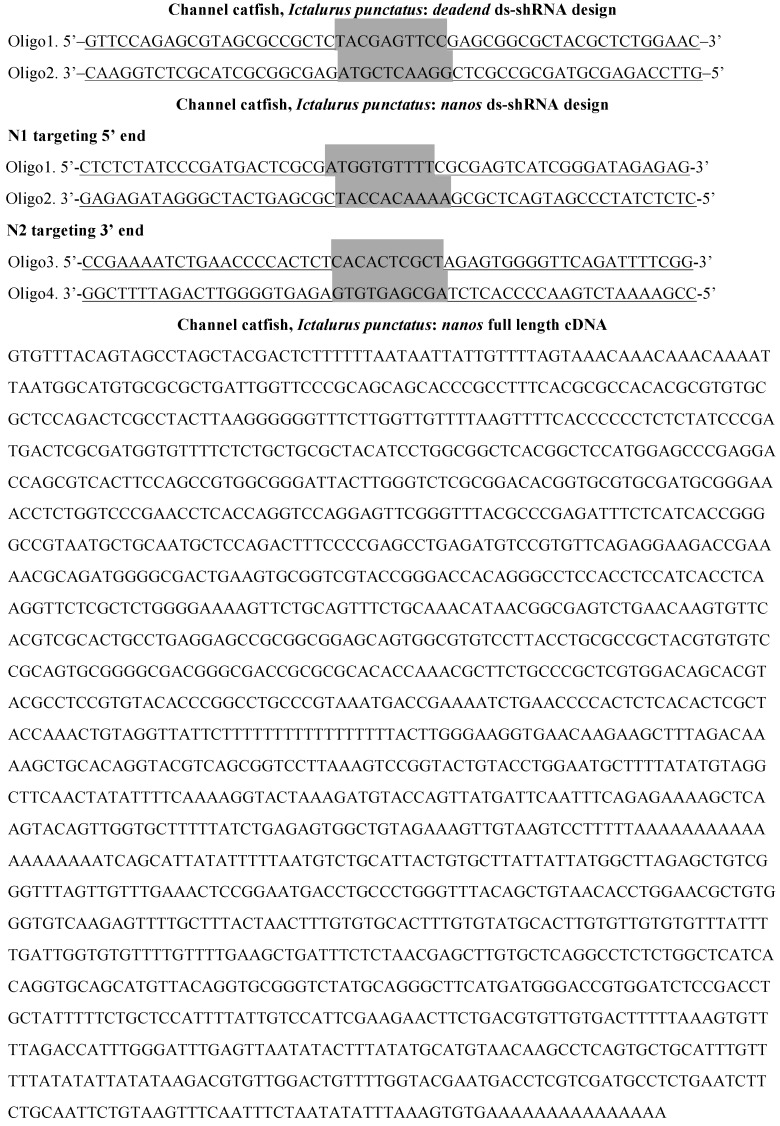
Primordial germ cell knockdown sequences for channel catfish, *Ictalurus punctatus*. Loops were in grey shade, stem sequences are underlined. Channel catfish *nanos* full-length cDNA was sequenced by Su et al. [32].

Channel catfish full-length *nanos* cDNA and *dead end* sequences were known (GenBank: KM874264.1 and KM874265.1). Based on these sequences, three RNAi and one cDNA sequence were ligated into the knockdown constructs targeting the marker genes (Figure 10). The knockdown strategies included: (1) a ds-sh RNA targeting the 5′ end of channel catfish *nanos* gene (N1); (2) a ds-sh RNA targeting the 3′ end of channel catfish *nanos* gene (N2); (3) a full length cDNA sequence of channel catfish *nanos* gene to overexpress *nanos* (cDNA); and (4) a ds-sh RNA targeting channel catfish *dead end* gene (DND). Except for the nanos cDNA sequence, all other constructs have a short hairpin structures and double stranded RNAs.

**Figure 11:**
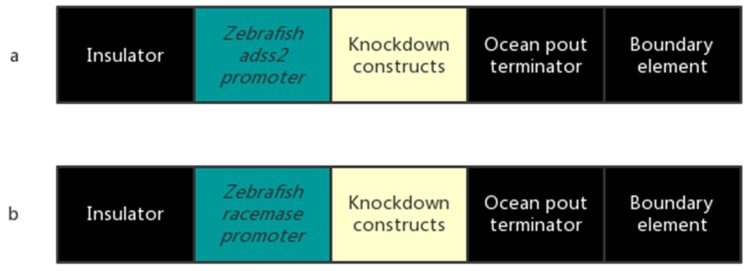
Primordial germ cell knockdown constructs for the sterilization of channel catfish, *Ictalurus punctatus*. (**a**) ADSS: The zebrafish *adenylosuccinate synthase 2* promoter is salt sensitive and was repressed by sodium chloride higher at 4 ppt. (**b**) RM: The zebrafish *racemase* promoter is salt sensitive and was repressed by sodium chloride higher at 4 ppt. Insulator, ocean pout terminator and boundary element were from FRMwg plasmid (GenBank: AF170915.1).

These constructs were designed and built as Tet-off-like systems (Figure 11). The constructs were synthesized at Genscript Biotech Corporation (Piscataway, NJ, USA).

### 4.2. P_1_ Fish

Plasmids with the transgene of interest were cloned into Invitrogen Top 10 *E. coli* cells, according to the recommended protocols by Invitrogen (user guide 280126, Invitrogen, Carlsbad, CA, USA). After the appropriate period of cloning cell culture, plasmid DNA was extracted using the Qiagen maxi-prep kit (Cat No./ID: 12163) and linearized with SfiI (20,000 units/mL, BioLabs, Ipswich, MA, USA). Plasmids were then purified using the phenol-chloroform-ethanol method and quantified using Thermo scientific Nanodrop2000**^®^** (Thermo Fisher Scientific, Waltham, MA, USA). Transgenes were transferred into one-cell, fertilized eggs using electroporation with a Baekon 2000 macromolecule transfer system (Baekon, Inc., Saratoga, CA, USA) to generate the P_1_ brood stock in the current experiment. Electroporation parameters were set at 6 kV, 2^7^ pulses, 0.8 s burst, 4 cycles, 160 μs [28,32]. Non-contact mode of electroporation with the electrode 1–2 mm above the buffer was applied. This procedure generated the P_1_ brood stock used in the current experiment. 

### 4.3. Artificial Spawning and Embryo Culture

During catfish spawning seasons when water temperature was 25 °C or higher, gravid females and well developed males were injected or implanted with luteinizing hormone releasing hormone analogue (LHRHa) to induce the ovulation of eggs and sperm with a priming dose of 30 μg·kg^−1^ followed by a resolving dose of 150 μg·kg^−1^ dose 12 h later [43,44]. When females began to ovulate they were anesthetized in 200 mg·L^−1^ tricaine methanesulfonate (Finquel: MS-222) with equal parts of NaHCO_3_ to maintain neutral pH for 10 min and then rinsed with freshwater [45]. Eggs were then hand stripped into a metal pan greased with vegetable shortening. 

P_1_ males and females putatively transgenic with the same transgene constructs were used to produce F_1_ offspring. Since P_1_ transgenic fish are always mosaic and usually mosaic by tissue [28], “transgenic” brood stock were identified based on the presence of the transgene in their F_1_ progeny. Thus, the initial incubation and treatments for the F_1_ were blind in regards to transgenic and non-transgenic groups. Testes were collected from euthanized males with well-developed secondary characteristics. Sperm were collected and then diluted with 0.9% saline at a 1:9 sperm: saline ratio. 

For fertilization, 2–3 mL diluted sperm solution was added to about 300 eggs then mixed. Enough pond water to submerse the eggs was added for gamete activation and fertilization. After 5 min, the pans with embryos were transferred to a trough with flow-through water and calcium for 1 h for water hardening. Embryos for treated groups (*T*) were then transferred into aerated tubs with 5 L modified Holtfreter’s solution (NaCl 4 g, NaHCO_3_ 0.2 g, KCl 0.05 g, MgSO_4_ 333 μL (300 g in 500 mL), CaCl_2_ 333 μL (150 g in 500 mL), pH: 7–7.5 in 1.0 L Dechlorinated water) [46]. Embryos from untreated groups (*U*) were transferred into flow through troughs fitted with aeration and paddlewheels for incubation.

The repressor treatment was 4 ppt sodium chloride, and, in this case, incubation was in tubs holding approximately 100 embryos and the treatment was continuous for the first 6 days of embryonic development. Incubation solutions were changed every 24 h. 

### 4.4. Embryo Sample Collection and RNA Extraction

Triplicate samples of embryos were collected at 24, 48 and 120 h post-fertilization (hpf) for both T and U groups. A total of 10–15 embryos were collected per replicate, paper towel dried, placed into 1.5 mL Eppendorf tube and immediately frozen in liquid nitrogen. Samples were then stored in −80 °C for future RNA or DNA extraction. Non-transgenic samples were also collected as a control.

Samples were ground into powder and dissolved in Invitrogen TRIzol**^®^** reagent (catalog # 15596-018), and RNA was then extracted following the manufacturer’s instructions. The quality and concentration of all the RNA samples were checked by both gel electrophoresis and with a Thermo scientific Nanodrop2000**^®^**. All extracted samples had an A_260/280_ ratio greater than 1.8, and were diluted to around 500 ng·μL^−1^.

### 4.5. PCR

PCR was used to identify transgenic positive and negative F_1_ families. Embryos were sampled from all families at 24 hpf, and DNA was extracted using QIAGEN^®^ buffer set, following the procedures of the QIAGEN^®^ genomic DNA handbook (2012), then purified and diluted to work as PCR template. Three sets of 3 pooled embryos were tested for each family for possession of transgenes. Eight pairs of primers were designed to amplify specific segments of the FRMwg plasmid bone structure sequence corresponding to the two different promoter sequences and the four different knockdown sequences (Table 4). PCR products were gel electrophoresed to identify positive families.

### 4.6. Real-Time PCR 

RNAs were reverse transcribed into cDNAs by iScript Synthesis Kit (Bio-Rad, catalog #: 170-8891). Each reaction consisted of a total volume of 10 μL containing 4.0 μL iScript reaction mix, 1.0 μL iScript reverse transcriptase, 500 ng RNA template, and water to reach the 10.0 μL volume. The reaction followed the protocol: 5 min at 25 °C, 30 min at 42 °C, 5 min at 85 °C. cDNAs were then diluted to 200 ng·μL^−1^.

qPCR was conducted with a C1000 Thermo-Cycler (Bio-Rad, USA) by using the SsoFast^TM^ EvaGreen^®^ supermix kit (Bio-Rad, catalog: #172-5201). Reactions were performed in a 10 μL total reaction volume (9.0 μL mix and 1.0 μL cDNA). The mix contained 1.0 μL of each primer (5 μmol·μL^−1^), 4.0 μL SsoFast EvaGreen supermix, and 3.0 μL RNase/DNase-free water. The same cycling conditions were used for all the tested samples: (1) denaturation, 95 °C for 30 s; (2) 40 cycles of 95 °C for 5 s, and 57 °C for 15 s; followed by (3) melting curve analysis, 5 s at 65 °C, then up to 95 °C with a 0.1 °C temperature increase every second. The mRNA levels of all the samples were normalized to the levels of the non-transgenic control sample of the same time point. Ribosome 18 s mRNA was used as a reference gene. Crossing-point (Ct) values were exported into Excel sheet from Bio-Rad CRX Manager (Version 1.6.541.1028, 2008). Su et al. [32] designed primers used for qPCR for the current experiment.

### 4.7. Spawning Evaluation

From 2012 to 2015, 99 P_1_ females electroporated with (exposed to) the salt sensitive sterilization constructs as one-cell embryos were injected with LHRHa to induce spawning or were evaluated for spawning readiness. A total of 58 were from the untreated group and 41 were from the treated group (Table 1). The spawning rates were recorded by transgenic treated and untreated, 2 promoter and 4 knockdown strategies, resulting in 16 groups in total. The records were then summarized and analyzed to reveal the spawning rate differences between knockdown strategies, between promoters and between treated and untreated groups.

### 4.8. Statistical Analysis

Fisher’s Exact Test and Fisher’s Multi-treatment Exact Test were used to compare rates of sexual maturity and spawning between and among the different treatment groups. REST (Relative Expression Software Tool) [47], using Pairwise Fixed Reallocation Randomization Test, was applied to compare the RNA expression level of the three PGC migration marker genes (*nanos, vasa and dead end*) among the treated group, untreated group and non-transgenic group and 18S was used as reference gene for this evaluation. Group means were used to express differences in expression between untreated, non-transgenic and treated samples. Statistical significance was performed by randomization test with 2000 randomizations per test (*p* < 0.05).

## Figures and Tables

**Table 1:** Spawning rates of P_1_ female channel catfish (*Ictalurus punctatus*) electroporated with constructs designed to disrupt primordial germ cell migration and then either untreated or treated with sodium chloride expected to repress the expression of the transgene. Construct components were: ADSS, zebrafish *adenylosuccinate Synthase 2 (adss2)* promoter; RM, zebrafish *racemase* promoter; N1, 5’ end ds-shRNA targeting channel catfish *nanos* gene; N2, 3’ end ds-shRNA targeting channel catfish *nanos* gene; cDNA, full-length cDNA sequence of channel catfish *nanos* gene; DND, ds-shRNA targeting channel catfish *dead end* gene. Treated embryos were repressed with 4 ppt sodium chloride (NaCl). * The difference between untreated and treated spawning rates of fish possessing that construct was significant (*p* < 0.05, Fisher’s Exact Test). ** Spawning rate of pooled untreated and treated P1 was different (*p* = 0.0002, Fisher’s Exact Test). (Typical induced spawning rates of non-transgenic catfish unexposed to PGC knockdown constructs is approximately 90% [30]).

Constructs	Untreated			Treated		
	Spawned	Total	%	Spawned	Total	%
ADSSN1	1	4	25	-	-	-
ADSSN2 *	2	8	25	4	4	100
ADSScDNA	8	13	62	8	8	100
ADSSDND	6	8	75	5	5	100
RMN1	7	9	78	8	8	100
RMN2	6	8	75	8	10	80
RMcDNA	2	3	67	3	3	100
RMDND	2	5	40	2	3	67
Total **	34	58	59	38	41	93

**Table 2:** Spawning rates of P_1_ female channel catfish (*Ictalurus punctatus*) electroporated with constructs designed to disrupt primordial germ cell migration and then either untreated or treated with sodium chloride expected to repress the expression of the transgene. Construct components were: ADSS, zebrafish *adenylosuccinate Synthase 2* (*adss2*) promoter; RM, zebrafish *racemase* promoter; N1, 5’ end ds-shRNA targeting channel catfish *nanos* gene; N2, 3’ end ds-shRNA targeting channel catfish *nanos* gene; cDNA, full-length cDNA sequence of channel catfish *nanos* gene; DND, ds-shRNA targeting channel catfish *dead end* gene. Asterisk (*) behind the construct name means the difference untreated and treated spawning rates of fish possessing that construct was significant (*p* < 0.05, Fisher’s Exact Test). Treated embryos were repressed with 4 ppt sodium chloride (NaCl). Ratio T/U: treated spawning rate/ untreated spawning rate.

Constructs	Untreated			Treated			Ratio of T/U
	Spawned	Total	%	Spawned	Total	%	
ADSS *	17	33	52	17	17	100	1.94
Rm	17	25	68	21	24	88	1.29
N1	8	13	62	8	8	100	1.61
N2 *	8	16	50	12	14	86	1.72
cDNA	10	16	63	11	11	100	1.59
DND	8	13	62	7	8	82	1.32

**Table 3:** Interaction between *nanos*, *vasa* and *dead end*. From the current experiment, assuming interaction rather than off-target knockdown, knockdown of *dead end* resulted in downregulation of *nanos* and *vasa*, knockdown of *nanos* resulted in downregulation of *vasa* and *dead end*. *Dead end* can regulate *nanos* by inhibiting the activity of miR-430 and protect *nanos* from degradation [18,37]. *Dead end* might be involved in regulation of *dazl*, thus indirectly regulates the expression of *vasa* [39,41]. *Vasa* is required for promoting the translation of *nanos* [38]. *Nanos* regulates *vasa* by suppressing the expression of *dazl* [39,40]. The potential mechanism of *nanos* regulating the expression of *dead end* is still unknown.

	Regulator Gene	*Nanos*	*Vasa*	*Dead End*
Responder Gene				
*Nanos*			*Vasa* is required for promoting translation of *nanos*	*Dead end* protects *nanos* from being degraded by miR-430 in germ cells
*Vasa*		*Nanos* suppresses *dazl*, *dazl* regulates *vasa* expression		*Dead end* potentially regulates *dazl*, *dazl* regulates *vasa* expression
*Dead end*		*Nanos* potentially regulates *dead end*, mechanism unknown	No known regulation found	

**Table 4:** List of primers used for PCR identification of transgenic channel catfish (*Ictalurus punctatus*) for 8 primordial germ cell knockdown constructs. Construct components were: ADSS, zebrafish *adenylosuccinate synthase 2* (*adss2*) promoter; RM, zebrafish *racemase* promoter; N1, 5′ end ds-shRNA targeting channel catfish *nanos* gene; N2, 3′ end ds-shRNA targeting channel catfish *nanos* gene; cDNA, full-length cDNA sequence of channel catfish *nanos* gene; DND, ds-shRNA targeting channel catfish *dead end* gene.

Primers	Sequence (5′–3′)
Forward primer for insulator	ACACTGTCCTTGGTATCAGCA
Reverse primer for insulator	ACACCTCCTTGATCCTGTGC
Forward primer for ocean pout terminator	TGCCCAAATTTCCTGCCTGA
Reverse primer for ocean pout terminator	TGCCTGTGAGGGTGACAAAA
Forward primer for *adss2* promoter	CCGCCAAAATACGACTG
Reverse primer for *adss2* promoter	TAAGCGAGGGAACAACG
Forward primer for *racemase* promoter	TACAACACCCACAATCCC
Reverse primer for *racemase* promoter	CACCGTAACAAAGAAACTCC
Forward primer for N1	ATGACTCGCGATGGTGTTTT
Reverse primer for N1	CATACTGGGGTGTTTGCTGA
Forward primer for N2	CACTCGCTAGAGTGGGGTTC
Reverse primer for N2	TCATACTGGGGTGTTTGCTG
Forward primer for cDNA	GAACCCCACTCTCACACTCG
Reverse primer for cDNA	AGACCCGCACCTGTAACATG
Forward primer for DND	TAGCGCCGCTCTACGAGT
Reverse primer for DND	TCATACTGGGGTGTTTGCTG
